# The multi-disciplinary management of complex congenital and acquired tracheo-oesophageal fistulae

**DOI:** 10.1007/s00383-018-4380-8

**Published:** 2018-11-03

**Authors:** H. S. Thakkar, R. Hewitt, K. Cross, E. Hannon, F. De Bie, S. Blackburn, S. Eaton, C. A. McLaren, D. J. Roebuck, M. J. Elliott, J. I. Curry, N. Muthialu, P. De Coppi

**Affiliations:** 1 Neonatal and Paediatric Surgery, Great Ormond Street Hospital, London, UK; 2 Department of Otolaryngology, Great Ormond Street Hospital, London, UK; 3 Tracheal Team, Great Ormond Street Hospital, London, UK; 4 General Surgery Resident, KU Leuven, Leuven, Belgium; 5 Stem Cells and Regenerative Medicine Section, DBC, University College London, London, UK; 6 Department of Radiology, Great Ormond Street Children’s Hospital, London, UK; 7 Department of Cardiothoracic Surgery, Great Ormond Street Hospital, London, UK

**Keywords:** Recurrent tracheo-oesophageal fistula, Button battery, Bronchoscopy, Thoracotomy, Cardio-pulmonary bypass

## Abstract

**Aim of the study:**

Complex tracheo-oesophageal fistulae (TOF) are rare congenital or acquired conditions in children. We discuss here a multidisciplinary (MDT) approach adopted over the past 5 years.

**Methods:**

We retrospectively collected data on all patients with recurrent or acquired TOF managed at a single institution. All cases were investigated with neck and thorax CT scan. Other investigations included flexible bronchoscopy and bronchogram (B&B), microlaryngobronchoscopy (MLB) and oesophagoscopy. All cases were subsequently discussed in an MDT meeting on an emergent basis if necessary.

**Main results:**

14 patients were referred during this study period of which half had a congenital aetiology and the other half were acquired. The latter included button battery ingestions (5/7) and iatrogenic injuries during oesophageal atresia (OA) repair. Surgical repair was performed on cardiac bypass in 3/7 cases of recurrent congenital fistulae and all cases of acquired fistulae. Post-operatively, 9/14 (64%) patients suffered complications including anastomotic leak (1), bilateral vocal cord paresis (1), further recurrence (1), and mortality (1). Ten patients continue to receive surgical input encompassing tracheal/oesophageal stents and dilatations.

**Conclusions:**

MDT approach to complex cases is becoming increasingly common across all specialties and is important in making decisions in these difficult cases. The benefits include shared experience of rare cases and full access to multidisciplinary expertise.

## Introduction

Complex tracheo-oesophageal fistulae (TOF) are rare conditions in children [1]. Most often these occur after repair of congenital oesophageal atresia (OA) with a distal TOF (C type). Primary repair has a re-fistulisation rate of 3–5% [1, 2]. Complex TOFs can also be a result of oesophageal injury by ingestion of caustic fluids or button batteries [3, 4].

The surgical repair of complex recurrent TOF or acquired lesions is challenging for a number of reasons. First, the diagnosis itself can be difficult and often requires oesophageal contrast studies and endoscopies to confirm and intubate the fistula. The literature suggests that a prone oesophagogram whilst withdrawing an NG tube is the most sensitive investigation with the fewest false negatives [5]. The most sensitive test in our experience has been to perform bronchography with bronchoscopic (B&B) probing of the fistula pit.

The quality of tissues may present a surgical challenge, particularly in acquired injuries where the defects tend to be larger and the tissues friable with areas of ischaemia or even frank necrosis. In recurrent TOF the scarring due to previous surgery also makes the access and safe mobilisation of the trachea and oesophagus more difficult, increasing the risk of intra- and post-operative complications.

Different approaches for managing these fistulae have been described: endoscopic use of glue for small defects [6], standard thoracotomy with a variety of adjuncts such as interposition flaps [7] (pericardium, pleura), early abandonment of the oesophagus in preference of tracheal preservation and median sternotomy with or without the use of cardiopulmonary bypass (CPB) for the more complex cases [8, 9].

Following a recent increase in tertiary referrals of complex TOF cases to our institution, a multidisciplinary (MDT) approach has been adopted so as to treat every patient on an individual basis. In this paper, we discuss our MDT approach based on the case series of complex TOFs treated in our institution over the past 5 years.

## Methods

We retrospectively collected data on all patients with recurrent or acquired TOF managed at Great Ormond Street Hospital from January 2013 till July 2018. All patients were tertiary referrals from other UK or international surgical centres. All cases were investigated with contrast CT scan of the neck and thorax. Other investigations included B&B, MLB and oesophagoscopy.

All cases were subsequently discussed in our MDT meeting (Table 1). We followed a 3 “Ds” approach—*D*iagnosis, *D*iscussion and *D*ecision-making. Those present at our MDT include a cardiothoracic surgeon, diagnostic radiologist, ENT surgeon, general paediatric surgeon, intensive care physician, interventional radiologist and radiographer, respiratory physician and specialist nurses. We have specifically incorporated the experience of our specialist nurses into this MDT setting to create a complex aero-digestive team. Decisions on the approach to be used in each case were based on several factors. Size, position of defect in the trachea and aetiology were the most important factors when considering whether CPB was required for repair or whether an endotracheal tube could be safely placed distal to the TOF allowing conventional ventilation. Size of defect also determined whether complete circumferential control of the trachea may be needed for division and repair of the trachea—necessitating a sternotomy approach. Surgical history of repeated thoracotomy was also a relative indication for sternotomy.

**Table 1:** MDT approach to complex/acquired TOF

Diagnosis	Tertiary referral received	History reviewed External imaging reviewed Transfer to appropriate ward/intensive care	
Discussion	Investigations	Contrast CT thorax and neck Flexible bronchoscopy and bronchography and oesophagogram Airway endoscopy +/- oesophagoscopy	
	MDT discussion Cardiothoracic surgery Diagnostic radiologist ENT General surgery Intensive care physician Interventional radiologist Radiographer Respiratory physician Specialist nurses	Questions considered History—previous surgery, approach, etc. Co-morbidities—especially cardiac Defect Size Position in trachea Quality of tissue Possible repair possible—primary repair, slide or patch tracheoplasty	
Decision	Repair of cardiopulmonary bypass	Repair via thoracotomy	Tissue engineering/experimental

Fisher’s test and chi-squared tests were used for statistical analysis with *P* < 0.05 considered statistically significant.

## Results

14 patients were referred during this study period of which half had a congenital aetiology (C-TOF) and the other half acquired (A-TOF). Two patients were referred from outside the UK, with the remaining referred from other UK tertiary paediatric surgical centres. Table 2 summarises the patient demographics, medical history, reasons for referral to our institution, the nature of surgery undertaken at our centre and their outcomes. The aetiology for the majority of acquired fistulae was button battery ingestion with two iatrogenic cases from repair of oesophageal atresia.

**Table 2:** Summary of patients referred to our service

Gender	Primary diagnosis	Surgery at referring institution (age in months)	Complication leading to referral	Surgery at GOSH (age in months)	Surgery on CPB/time (mins)	Complications till date	Last follow-up
Congenital (*n* = 7)							
M	Type C OA/TOF	Thoracoscopic repair of OA/TOF (neonatal), thoracoscopic repair of recurrence (9 m), open repair of further recurrence (10 m)	Persistent recurrent fistula	Re-do aortopexy (11 m), thoracotomy and repair of persistent recurrent fistula (13 m)	Yes (113 min)	Further recurrent TOF—repaired through sternotomy on CPB (55 m) using Gortex patch, repair of tracheal posterior wall dehiscence on CBP (61 m)	2017
M	Type C OA/TOF	OA/TOF repair (neonatal), thoracotomy and repair of recurrent fistula (4 m), tissue glue for further recurrence (65 m)	Persistent recurrent fistula	Thoracotomy and repair of persistent recurrent fistula (68 m)	No	Nil	2018
M	Type C OA/TOF	Thoracotomy and repair of OA/TOF (neonatal), Thoracotomy and repair of recurrent TOF (1 m), Further recurrence and treatment with diathermy + glue (38 m)	Persistent recurrent fistula	Sternotomy, repair of persistent fistula and slide tracheoplasty (38 m)	Yes (91 min)	Nil	2016
F	Type C OA/TOF Type IV laryngeal cleft	Ligation of TOF + gastrostomy (neonatal), Jejunostomy for recurrent TOF (2 m)	Recurrent TOF	Tracheostomy, Thoracotomy and repair of recurrent TOF and oesophageal atresia (5 m), Two-stage repair of laryngeal cleft repair (11 m)	No	Recurrent laryngeal cleft—repaired (14 m)—further recurrence awaiting more surgery	2016
F	Type C OA/TOF and duodenal atresia	Ligation of TOF, division of TOF and primary OA repair (neonatal), oesophagostomy for leak, duodenal atresia repair and gastrostomy (1 m), two-stage oesophago-jejunal Roux-en-Y reconstruction (13 m, 23 m)	Oesophago-jejunal cervical fistula	Initial stent placement across the fistula by interventional radiology followed—localised abscess formation—CT scan and bronchogram showed recurrent TOF—laparoscopic-assisted gastric transposition and neck approach for disconnection of oesophago-jejunal anastomosis, re-direction of Roux limb of jejunum to fashion Roux-en-Y jejunostomy (36 m)	No	Oesophago-gastric anastomotic leak managed conservatively	2018
M	Type C OA/TOF	OA/TOF repair (neonatal), thoracotomy and repair of recurrent fistula (2 m)	Persistent recurrent fistula	Sternotomy, repair of recurrent fistula with autologous pericardial patch tracheoplasty (6 m)	Yes (146 min)	Death from complete tracheal dehiscence with necrosis	N/A
M	Type D OA/TOF	Thoracotomy and repair of OA/TOF (neonatal)	Recurrent TOF, left vocal cord palsy	Thoracotomy and repair of recurrent TOF (5 m), thoracoscopic aortopexy (6 m)	No	Bilateral poor vocal cord movement	2018

Gender	Age at presentation to local (months)	Primary diagnosis	Complication leading to referral	Surgery at GOSH (age in months)	Surgery on bypass	Complications till date	Last follow-up
Acquired (*n* = 7)							
F	23	Button battery ingestion	Acquired TOF	Sternotomy, direct repair of oesophagus, gastrostomy and slide tracheoplasty (23 m), laparoscopic-assisted gastric transposition (45 m), stem-cell tracheal transplant (47 m)	Yes (124 min)	Thoracotomy for recurrent fistula, excision of mid-oesophagus and oesophagostomy (29 m), re-do sternotomy and tracheal repair on CPB (46 m)	2018
M	20	Button battery ingestion	Acquired TOF	Sternotomy, direct repair of the oesophagus and slide tracheoplasty (20 m)	Yes (101 min)	Post-operative mediastino-cutaneous fistula, recurrent fistula repaired with autologous pericardial patch tracheoplasty and direct repair of the oesophagus (26 m)	2018
M	3	Long-gap OA	Iatrogenic tracheal injury at initial surgery repaired during thoracotomy for delayed primary repair of OA but later development of acquired TOF	Excision of trachea-oesophageal fistula, autologous pericardial patch tracheoplasty, closure of proximal and distal oesophageal stumps (3 m), laparoscopic- assisted gastric transposition and jejunostomy (16 m)	Yes (135 min)	Laparoscopic ligation of distal oesophagus for recurrent fistula, gastrostomy (4 m), air leak requiring tracheal repair under bypass (4 m)	2018
F	12	Button battery ingestion	Acquired TOF	Near total oesophagectomy, cervical oesophagostomy, gastrostomy, autologous pericardial patch tracheoplasty (12 m), laparoscopic-assisted gastric transposition (22 m)	Yes (110 min)	Transient bilateral vocal cord palsy–tracheostomy (23 m) now decannulated	2018
M	11	Button battery ingestion	Acquired TOF	Sternotomy, autologous pericardial patch tracheoplasty, direct repair of the oesophagus and formation of gastrostomy	Yes		2018
M	3	Long-gap OA	Iatrogenic injury to the left main bronchus repaired during thoracotomy for delayed primary repair of OA but later development of acquired TOF—attempted repair locally with muscle flap	Sternotomy, left main bronchus patch repair and direct repair of the oesophagus	Yes (180 min)		2018
M	18	Button battery	Acquired TOF	Sternotomy, autologous pericardial patch tracheoplasty, direct repair of the oesophagus (18 m)	Yes (118 m)		2018

**Fig. 1:**
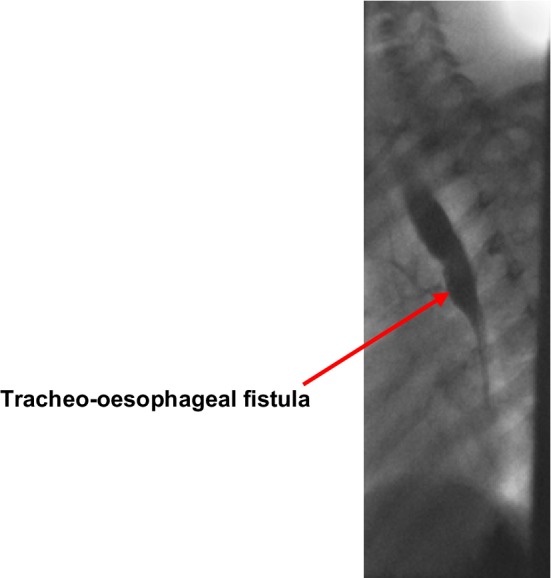
A lateral bronchogram image of a patient with a persistent, congenital recurrent fistula—background of OA/TOF

**Fig. 2:**
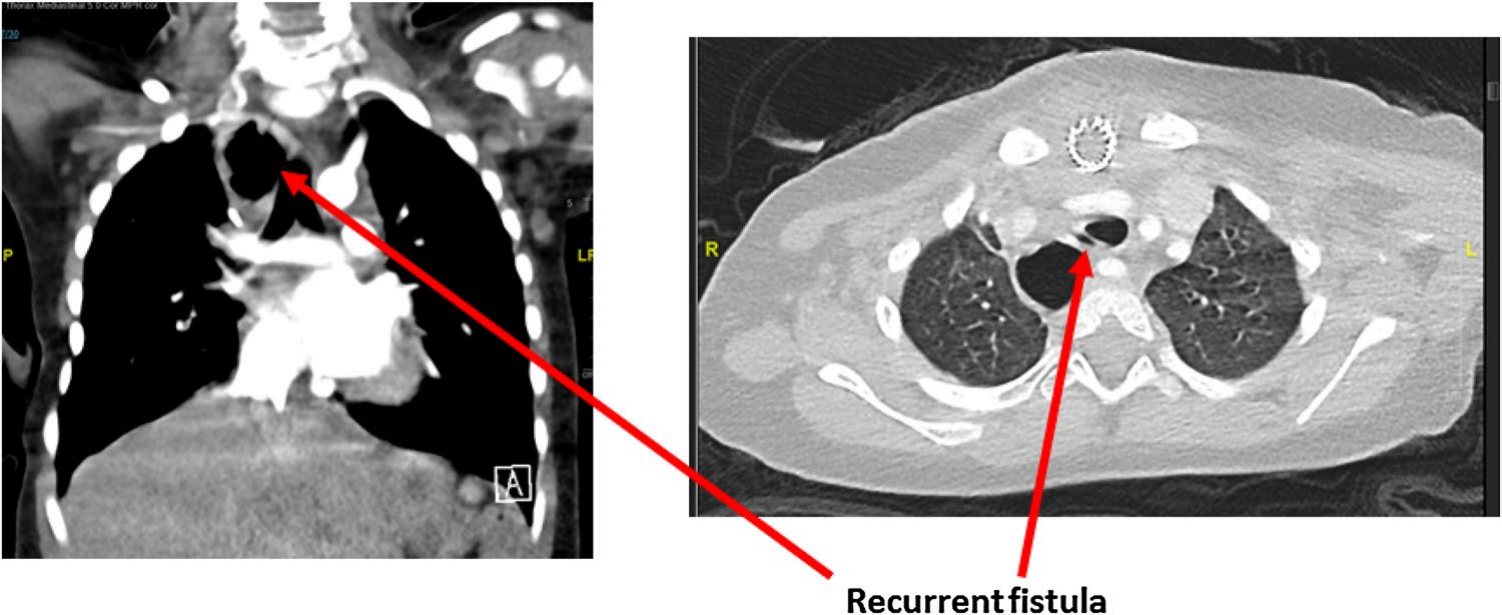
A CT scan demonstrating a complex cervical tracheo-oesophageal fistula in a patient with previous jejunal interposition—background of OA/TOF, duodenal atresia

**Fig. 3:**
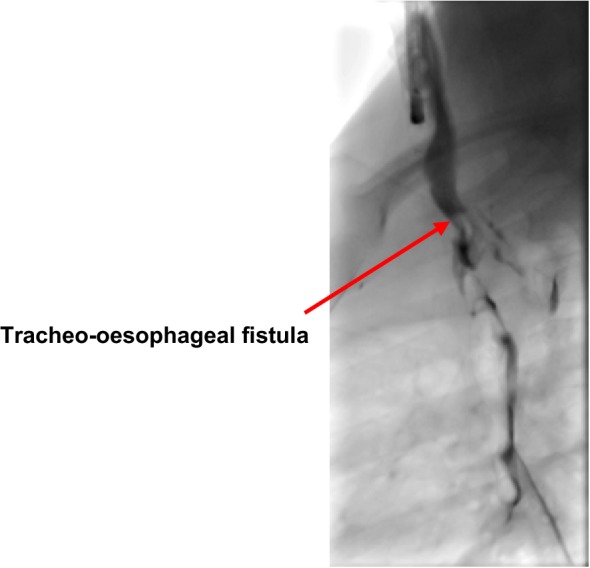
A lateral bronchogram image of a patient with an acquired TOF post-button battery ingestion with corresponding endoscopic images

**Fig. 4:**
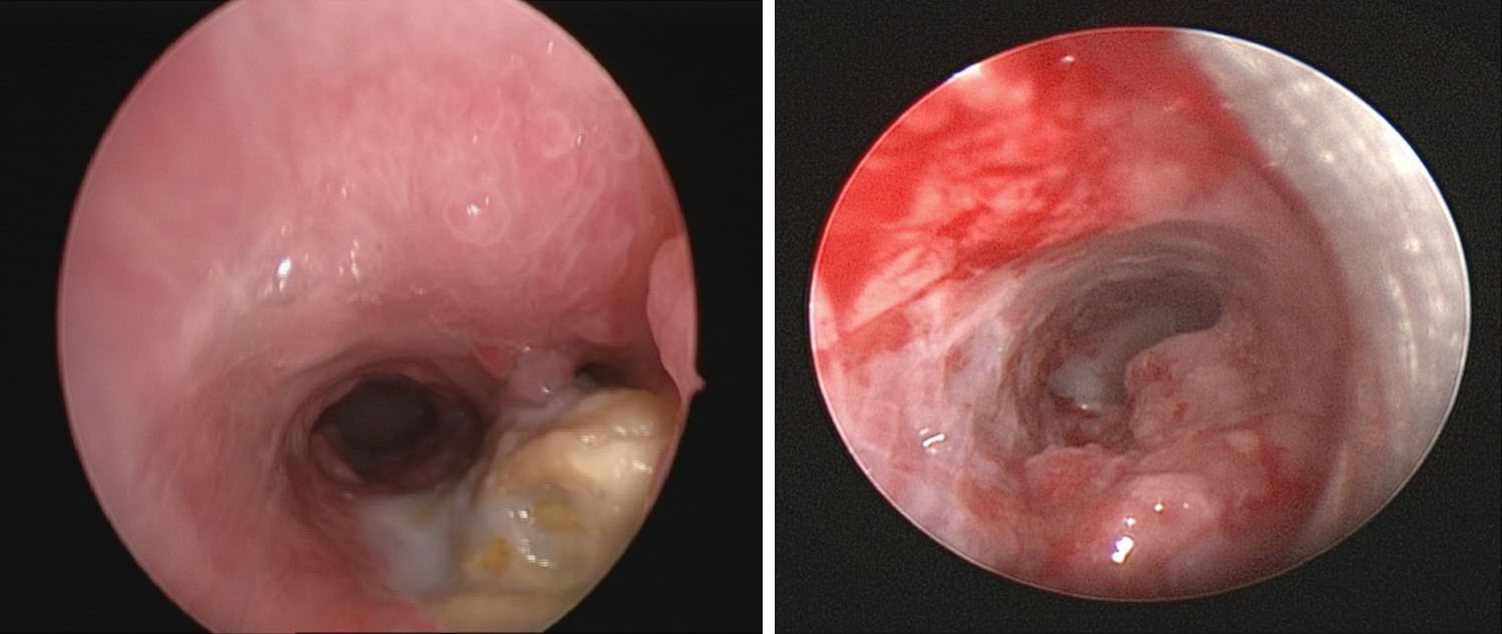
Endoscopic images of two patients with fistulae post-button battery ingestion

Figures 1, 2, 3 and 4 illustrate bronchograms and endoscopic images of patients in our series with complex fistulae.

### Surgical approach

**Fig. 5:**
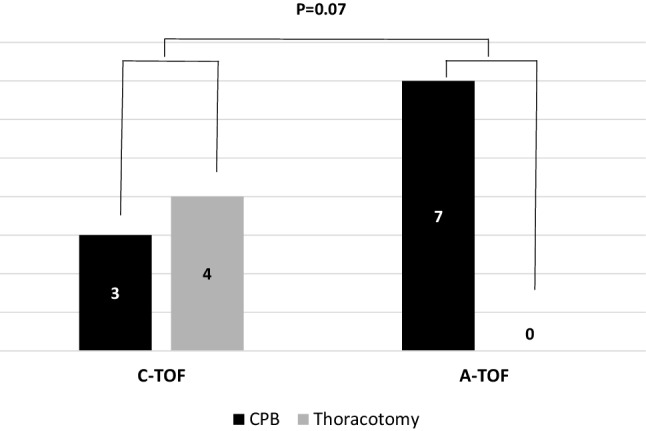
Surgical approach for the management of congenital and acquired fistulae

Figure 5 illustrates the surgical approach for each of the cases. Surgical repair was performed in all cases with CPB used in 3/7 (43%) cases of congenital recurrent fistulae and all cases (100%) of acquired fistulae. The median bypass time was 118 min (91–180 min).

### Oesophageal replacement

**Fig. 6:**
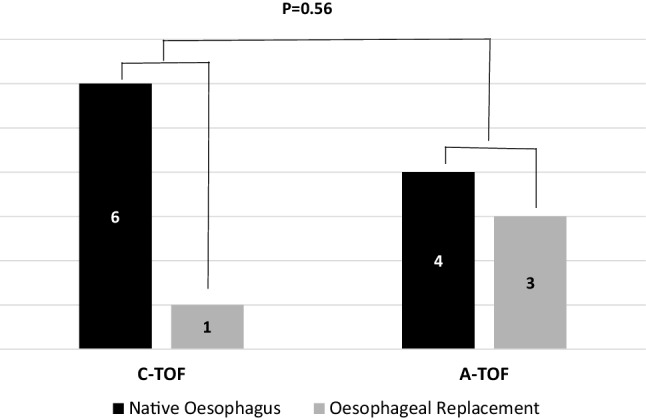
Retention of native oesophagus amongst patients with congenital and acquired fistulae

Major emphasis is made to preserve tracheal tissue at all costs for obvious reasons. We have also tried to adopt a conservative approach for the oesophagus when possible. In 4/14 (30%) of cases, it was sacrificed only after a second exploration. Figure 6 illustrates retention of the native oesophagus in our cohort of patients. When removed, patients received an oesophagostomy with a plan for future oesophageal replacement. Our preferred method for this has been laparoscopic-assisted gastric transposition.

### Complications and follow-up

**Fig. 7:**
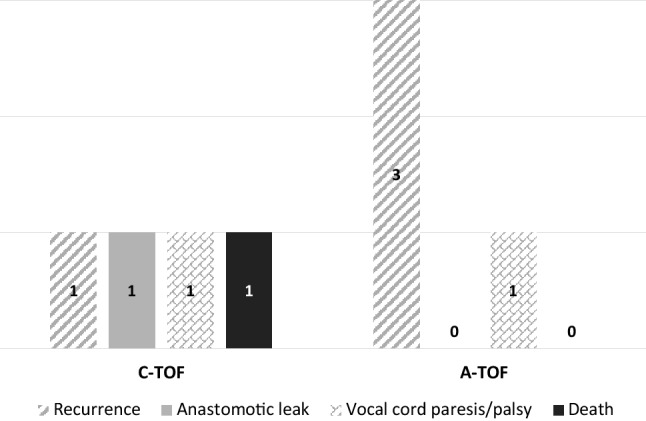
Surgical complications amongst patients with congenital and acquired fistulae

Surgery for complex TOFs is challenging and despite MDT discussion and careful surgical planning, minor or major complications in 8/14 (60%) patients exist (Fig. 7).

There was one death in a patient who initially underwent a thoracotomy and repair of OA/TOF at one referring institution. The infant developed a recurrent fistula at 2 months of age which was operated on again through a thoracotomy. Unfortunately, the fistula recurred again and the patient was transferred intubated and ventilated from paediatric intensive care to our institution. He underwent a repair of this fistula under CPB with an autologous pericardial tracheoplasty and direct oesophageal repair. This recurred again and further surgery was performed under CBP including an oesophagostomy and gastrostomy. One week later he suffered from an acute deterioration with left-sided tension pneumothorax. He was stabilised from this but continued to suffer from an ongoing air leak. This was explored through a sternotomy on extra-corporeal membrane oxygenation (ECMO) and after discovering tracheal necrosis with active infection the MDT decision was to not proceed any further.

**Fig. 8:**
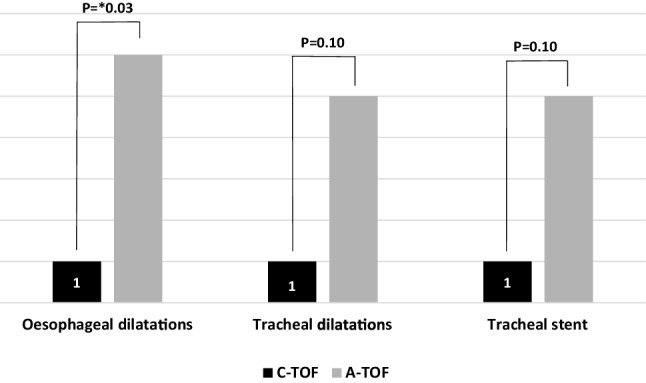
Ongoing management of patients following surgery

10 patients continue to receive surgical input at our institution with 3 patients now cared for at their original referring centre. Figure 8 summarises the treatments used for these patients as part of their ongoing management at our institution.

### Aerodigestive outcomes

The majority of all our patients are currently orally fed. 2/7 (30%) patients in the acquired group are having also need additional feeds via a gastrostomy or jejunostomy. From an airway perspective, none of our patients are currently receiving supplemental oxygen. 1/7 (14%) patient in the acquired group needed a tracheostomy for transient bilateral vocal cord palsy which has now been decannulated.

## Discussion

Complex cases of acquired and recurrent TOF are rare in children and as such require careful consideration of the best and safest approach to their management. At our institution, we have developed an MDT approach to these cases with individualised management directed towards their complex needs. Our patient cohort can essentially be divided into two cohorts: (1) congenital recurrent fistulae and (2) acquired cases largely from button battery ingestion. Our experience with the latter group has been more challenging with the outcomes similarly reflecting the complexity of the condition.

The incidence of recurrent congenital tracheo-oesophageal fistula has been estimated to be 3–14% [1]. The diagnosis of a recurrent fistula can be difficult and often requires a combination of radiological and endoscopic studies. The prone tube oesophagogram is a reasonable first test. If, however, this test is negative and high index of clinical suspicion exists, we would proceed to a B&B with probing of the fistula pit. The bronchoscopy can be flexible or rigid but should be performed with high-quality fluoroscopy in lateral projection and preferably biplane.

Whereas endoscopic management alone of these has been described [7], our preference has been to perform surgical repair because of the high risk of recurrence after conservative management. In a systematic review of 165 patients across 44 studies [5], the success rate of surgery was found to be 93.5% compared with 84% for endoscopic treatment. In our study, patients had either failed endoscopic management prior to their referral to our institution or they were too large to be suitable for this approach. For this reason, all patients in our study underwent surgical repair.

Ingested button batteries can cause severe and rapid injury to mediastinal structures. This problem has recently received a lot of media attention through several recent high-profile cases [10]. In the US, the National Poison Data System estimates an incidence of 6.3–15.1 button battery ingestions per million of the population annually [11]. In the UK, the Child Accident Prevention Trust and British and Irish Portable Battery Association have both launched recent campaigns highlighting the potential dangers to families. In our cohort of acquired fistulae, 5/7 (71%) of cases were due to button batteries and all these cases were managed surgically. Conservative management of such fistulae has also been reported [11]; however, we believe that this is not effective largely due to the friable nature of the tissue and the presence of ischaemia or necrosis. As a specialist referral centre, we also tend to see patients on the severe end of the spectrum which tends to skew our preference towards surgical management.

Several operative approaches have been described in the literature when approaching these complex fistulae [7–9]. The decision of which approach is most suitable is made within our MDT meetings with sufficient detailed imaging allowing a full understanding of the anatomy pre-operatively. We, therefore, perform a combination of cross-sectional imaging (CT thorax) and contrast studies or endoscopy of airway and oesophagus. The key factors to consider are the size and position of the fistula, quality of tissue, type of injury, previous surgery and co-morbidities.

The size and position of the fistula, in particular, are pertinent in determining whether ventilation will be possible using a distally placed endotracheal tube. The more distal the fistula the harder it becomes to reliably ventilate the patient. Tissue quality is also a problem after surgery for recurrent cases or battery injuries. It must also be remembered the fistulae will probably be made larger following dissection or debridement of necrotic tissue. Although it may be possible to safely ventilate these patients pre-operatively, this may change significantly following dissection of the defect.

In some patients that had a congenital aetiology with at least two previous recurrences, direct repair of the trachea or a more complex tracheoplasty required maximal control of ventilation and oxygenation. In those cases, we have, therefore, opted to perform the repair on CPB through a midline sternotomy as opposed to conventional thoracotomy [12]. Provenzano et al. [13] report utilising CPB in patients with distal TOF for which they favour a slide tracheoplasty repair, but do not describe in detail their decision-making process for this. Tibballs et al. have also demonstrated the need for CPB in the case of a huge relatively proximal TOF from a button battery injury in which adequate oxygenation could not be adequately achieved with conventional ventilation [14].

The complexity of the patients that have been referred to our institution is clearly evident from this report with our outcomes reflective of the severity of the conditions. As an institution, we often receive a select population of patients which have already previously failed conservative or surgical management.

There was one death (7%) in the study group in a patient with a persistent, recurrent congenital TOF who died from tracheal necrosis following four attempts at repair. Wang et al. in their series of 35 patients with a recurrent fistula report a mortality rate of 8.6% [1]. In their series, the patients died from chest infections/sepsis following surgery. In our patient, repeated surgery on the trachea led to eventual necrosis and despite a trial of ECMO the patient did not survive. One further patient in the congenital group referred with a persistent TOF developed a recurrence after surgery. This patient required surgery on CPB on two further occasions and has required a biodegradable tracheal stent with ongoing tracheal and oesophageal dilatations.

In the acquired group, 3/7 (43%) patients have had further recurrences. The high incidence of recurrence and leaks in our series is a result of several factors. First, the complexity of the defects, especially those at the carina makes successful primary repair more difficult. Second, large defects on the oesophageal end of the fistula are often repaired directly and are prone to leakage and subsequent fistulation due to the friable nature of the tissue.

Other complications encountered in our series were tracheal and oesophageal strictures that were treated regularly with dilatations. The acquired group, in particular, required significantly more oesophageal dilatations (86%) compared with the congenital group (14%). A similar experience has recently been reported in a series of patients from the Netherlands [15]. As already discussed, this is largely due to the damage caused to the oesophagus through pressure necrosis, chemical alkaline damage and generation of an electrical current [15].

In conclusion, the MDT approach to complex cases is becoming increasingly common across all specialties and is important in making decisions in these difficult cases. The benefits of such an MDT approach are well recognised at other leading centres dealing with a similar complex case mix [16]. When faced with such complex patients, taking an MDT approach enables holistic care to be delivered to the patient. Furthermore, each member of the team can draw on their experience and reciprocally the proficiency of the group is enhanced with each case faced collectively. We advocate early referral of these complex cases to centres where such expertise is available to offer the full range of treatment to patients.
